# Friend or foe? Carbon monoxide and the mitochondria

**DOI:** 10.3389/fphys.2015.00017

**Published:** 2015-02-03

**Authors:** Nils Schallner, Leo E. Otterbein

**Affiliations:** ^1^Department of Surgery, Beth Israel Deaconess Medical Center, Harvard Medical SchoolBoston, MA, USA; ^2^Department of Anesthesiology and Intensive Care Medicine, University Medical Center FreiburgFreiburg, Germany

**Keywords:** heme, heme oxygenase, bioenergetics, hemoprotein, CO-RM

## Physiology of carbon monoxide

The longstanding perception of the gas carbon monoxide (CO) as an odorless and colorless “silent killer” began to attract the attention of the public with the arrival of the industrial age in the beginning of the twentieth century (Douglas et al., 1912). In fact, carbon monoxide has been present in all societies since the discovery of fire, yet it was John Haldane in the early part of the twentieth century that declared CO a lethal poison based on his investigations of mine disasters. American Indians knew that in addition to warmth, gathering around a fire brought calming and tranquil effects, something we now attribute to neuroactive properties of the gas. Poisonings from exhaust certainly continue to pose significant problems, as it did in the coal mine explosions, but it remains unclear why the >500 other molecules that emerge from combustion, many of which are carcinogens, are largely ignored, yet pose just as great a risk as CO. It was not until the late 1960's that endogenous production of CO was discovered as a result of the catabolism of heme (Sjostrand, 1949; Coburn et al., 1963), suggesting a physiological role for this simple, diatomic gas. Decades after these findings were reported, investigators noted that levels of CO were significantly elevated in the exhaled breath of hospitalized patients (Vos et al., 2009; Cheng et al., 2010; James et al., 2010; Zhang et al., 2010). The illnesses were wide-ranging, yet it was clear that CO levels would decrease as the pathology resolved. How then can it be explained that CO is toxic if the body generates it physiologically and even more puzzling, generates more when in a compromised state? The answer may lie in the ancient organelle known as the mitochondria, an evolutionary endo-symbiont originating from proteobacteria whose singular responsibility is to generate energy for the cell. It relies principally on the presence of gases in the elegant transfer of electrons among the oxidases contained within its membranes.

The targets for CO are ostensibly clear. CO binds rapidly and with high affinity to heme-containing proteins such as hemoglobin, the mitochondria oxidases or the enzymes necessary for reactive oxygen species generation. CO competes with oxygen transport and cellular respiration and it is perhaps in this primitive symbiotic organelle, among the numerous hemoprotein complexes competing with the other bioactive gases including nitric oxide, oxygen, hydrogen sulfide and carbon dioxide that CO integrates itself and impacts cellular physiology. The body of evidence supporting a physiological role for CO is immense and continues to move forward as CO is being evaluated in ongoing clinical trials (www.clinicaltrials.gov, Identifier: NCT 01727167, 00094406, 00122694, 01214187, 01050712, 01050933, 01523548, and 00531856).

The endogenous generation of CO as described by Tenhunen et al. (1968) occurs through the enzymatic degradation of heme by the heme oxygenases, enzymes present in all cells that convert heme into biliverdin, iron and CO. Like CO, it has become undeniably clear that each catalytic product has important physiological functions beyond serving as byproducts. Two isoforms of heme oxygenase exist: heme oxygenase 1 (*Hmox-1*), which is expressed ubiquitously and is highly inducible by an array of stimuli, and the constitutive heme oxygenase-2 (*Hmox-2)* isoform, predominantly expressed in neurons, the testes, and the vasculature. Induction of HO-1 has proven to be a strong cytoprotectant while deficiency in HO-1 leads to aggravated disease states, even in humans (Poss and Tonegawa, 1997; Otterbein et al., 1999; Park et al., 2007; Tsuchihashi et al., 2007; Chen et al., 2009; Wang et al., 2009, 2012; Yin et al., 2010; Ferenbach et al., 2011; Ogawa et al., 2011; Zhang et al., 2012).

## CO as a therapeutic agent

There is compelling pre-clinical data proving the salutary effects of exogenous CO application. (Motterlini and Otterbein, 2010) CO has been shown to regulate immune responses (Freitas et al., 2006), cell survival (Song et al., 2003) and regeneration (Lin et al., 2009; Lakkisto et al., 2010) as well as proliferation (Wegiel et al., 2013). CO is homeodynamic in that it serves the need of the tissue. There are reports that it is both anti- and pro-inflammatory (Lee et al., 2007; Beckman et al., 2009), pro- and anti-apoptotic (Song et al., 2004; Vieira et al., 2008) and pro- and anti-proliferative (Otterbein et al., 2003; Kuramitsu et al., 2011). One of the primary sites in the body where CO is believed to be most toxic is the brain and this is based on weak studies with lack of rigor and proper controls. CO is clearly neuroprotective in various neuronal injury models (Vieira et al., 2008; Zeynalov and Dore, 2009; Wang et al., 2011; Yabluchanskiy et al., 2012; Schallner et al., 2013) and extensive safety trials in humans have been completed without a single sign of toxicity at carboxyhemoglobin levels of 12–15% (Mayr et al., 2005; Bathoorn et al., 2007). Most importantly, no negative influence on cognitive function was detected. Collectively, the clinical testing is safe with quantitative delivery of inhaled CO relative to body weight and independent of the respiratory rate has also been developed (Motterlini and Otterbein, 2010). The challenges of establishing CO as a gaseous pharmaceutical triggered an onslaught of research surrounding alternative routes of CO application. Carbon Monoxide Releasing Molecules (CO-RMs) emerged in 2002 pioneered by Roberto Motterlini (Motterlini et al., 2002). CO-saturated pegylated hemoglobins have emerged that also modulate inflammation and vaso-occlusion in murine models of sickle cell anemia (Belcher et al., 2013). These CO carriers, or pro-drugs, release CO following well-defined kinetics and have been characterized to deliver CO to target tissues in several *in vitro*(Clark et al., 2003; Motterlini et al., 2005; Bani-Hani et al., 2006; Megias et al., 2007; Urquhart et al., 2007) and *in vivo* (Tayem et al., 2006; De Backer et al., 2009; Tsoyi et al., 2009; Vadori et al., 2009) studies, exerting biological effects much like inhaled gas (Bani-Hani et al., 2006; Yabluchanskiy et al., 2012).

## CO and the mitochondria

Despite profound pre-clinical evidence of efficacy, the molecular mechanisms by which CO exerts its protective effects in a diverse array of animal models remains poorly characterized with numerous and confounding molecular targets described (Motterlini and Otterbein, 2010). The high affinity for heme makes any cellular heme-containing protein a potential target for CO, including soluble guanylate cyclase (sGC) (Verma et al., 1993; Schallner et al., 2013), NO-synthase (Zuckerbraun et al., 2003; Marazioti et al., 2011), NADPH oxidase (Taille et al., 2005) and NPAS-2 (Dioum et al., 2002) among a multitude of others. While a unifying signature is lacking, the single-most implicated target is the mitochondria. This seems paradoxical at first sight since inhibition of mitochondrial respiration via CO binding to components of the mitochondrial electron transfer chain, has been looked at as being responsible for the toxicity seen after CO poisoning. Against this dogma, however, CO exposure clearly influences cellular bioenergetics in the context of salutary effects, paradoxically increasing O_2_ bioavailability and consumption, which in turn reduces injury-related organ damage (Tsui et al., 2007; Lancel et al., 2009). CO increases mitochondrial generation of reactive oxygen species (Chin et al., 2007; Zuckerbraun et al., 2007) and mitochondrial biogenesis, (Suliman et al., 2007; Piantadosi et al., 2008) which likely go hand-in-hand to influence the vast array of cellular downstream targets that have been linked to the beneficial effects of CO (Motterlini and Otterbein, 2010). We speculate that CO alters oxygen sensing and exerts a “pseudo-hypoxic” state, providing a powerful cellular impact toward re-generation and increasing the cellular energy supply that leads to improved survival in the presence of cell stress and injury.

## Conclusions and perspective

The name mitochondria originated from the Greek “mitos” meaning thread and “chondros” meaning granule, which referred to their structural appearance. They were first called “bioblasts” which is perhaps a more accurate designation giving the impression of explosive behavior while generating critical energy for the cell. Mitochondria are comprised of lipid bilayers and proteins like other cellular compartments including the Golgi, endoplasmic reticulum and the nucleus. The mitochondria rely to a large extent on the interrelationships among the gases, primarily O_2_ and CO_2_. These gases serve as the fundamental molecules involved in the energy-transduction system that ultimately results in generation of life-sustaining ATP. It has become clear, however that O_2_ and CO_2_ are not alone in dictating cellular physiologic and pathophysiologic responses. Much like the complexities of signal transduction, gene regulation and metabolic pathways, the cellular gases CO and its sister gases NO and H_2_S are critically integrated into the function of mitochondria and therein the overall health of the organism.

### Conflict of interest statement

The authors declare that the research was conducted in the absence of any commercial or financial relationships that could be construed as a potential conflict of interest.
